# Multifocal Initiation of Spreading Depolarization by Generalized Tonic-Clonic Seizures

**DOI:** 10.3390/ijms27188429

**Published:** 2026-09-21

**Authors:** Maria Smirnova, Anna Karan, Tatiana Medvedeva, Lyudmila Vinogradova

**Affiliations:** Institute of Higher Nervous Activity and Neurophysiology, Russian Academy of Sciences, Butlerova Street 5A, 117485 Moscow, Russia; rymarik@gmail.com (M.S.); akartar.n@gmail.com (A.K.); golova93tanya@gmail.com (T.M.)

**Keywords:** spreading depolarization, epilepsy, generalized seizures, seizure termination, hippocampus, postictal EEG depression

## Abstract

Epileptic seizures are self-terminating events, yet the precise mechanisms underlying their cessation and the subsequent postictal suppression remain incompletely understood. Spreading depression (SD)—a traveling wave of intense depolarization and electrical silence—has emerged as a potential mechanism of focal seizure termination. Given that generalized tonic-clonic seizures involve widespread hyperexcitation of brain networks, we suggested that the seizures promote multifocal triggering of SD. Using a pentylenetetrazole seizure model and in vivo electrophysiology, we examined the occurrence of SD in different regions of the cortex and hippocampus in response to acute tonic-clonic seizures in freely behaving rats. We found that the seizures always induced SD within the first 20–40 s of ictal activity. SD was detected nearly simultaneously in all distant regions of the cortex and hippocampus. Most seizures were brief and culminated in SD. If the ictal discharge was long, cortical SD appeared mid-ictally. In the hippocampus, seizure-induced SD was partially blocked and spatially limited. Our findings support the idea that the awake brain rapidly responds to acute generalized seizures with multifocal initiation of SD in the cortex and hippocampus. We suggest that the seizure-induced SD contributes to seizure termination in the cortex but not in the hippocampus.

## 1. Introduction

Epileptic seizures are inherently self-terminating events, resolving spontaneously in the majority of clinical cases. Seizures are usually followed by the postictal state associated with generalized electroencephalographic (EEG) suppression and cognitive deficit. Elucidation of intrinsic mechanisms of seizure termination and postictal suppression remains an important unresolved question in epilepsy research. It is suggested that homeostatic feedback mechanisms are activated during the ictal discharge, promoting its termination and initiation of postictal events [1]. The mechanisms have been proposed to include neuronal exhaustion, the accumulation of extracellular potassium and adenosine, energy failure, network alterations, increased GABAergic inhibition, etc. [1,2]. However, the precise role and hierarchy of these factors are still under debate.

Recently, spreading depolarization (SD), a self-propagating wave of massive cellular depolarization, has been suggested to act as an intrinsic mechanism of seizure termination [3,4,5]. The pronounced influence of SD on gene expression, neuronal excitability, and circuit dynamics [6,7,8] suggests that it can contribute to the control of seizure activity. Substantial evidence indicates that SD is a reliable pathophysiological consequence of ictal activity. Co-occurrence of SD and seizures has been reported in patients with acute brain injury [9,10] and drug-resistant epilepsy [4,11]. Frequent association of cortical SDs with seizure termination has been shown in humans and animals [3,4,5,9], indicating the potential role of seizure-induced SD in cessation of ictal activity. Animal studies demonstrate that SD can terminate seizures and prevent their generalization within the brain [3,4,5].

A transient, profound silencing of local field potentials, the electrophysiological hallmark of SD, closely mirrors the postictal suppression observed clinically and positions SD as a plausible driver of the postictal state. Preclinical studies demonstrate that SD contributes to pathogenic mechanisms of sudden unexpected death in epilepsy, the most common cause of premature mortality in epileptic patients [12], postictal behavioral excitation [13], and postictal generalized EEG depression [14]. Thus, elucidation of SD involvement in mediating seizure termination is essential for understanding how the brain self-limits excessive excitability. However, SD is a commonly unmonitored event in epilepsy clinics, highlighting the importance of experimental studies for clarification of the role of SD in epilepsy.

Data from animal models demonstrate complex seizure–SD interplay: seizures either trigger [3,4,15] or suppress [3,16,17] SD, while SD can either stop [3,17] or provoke [17,18] seizures. Whether SD is a universal prerequisite for termination across different seizure types requires further investigation. Most experimental data have been obtained in models of focal cortical seizures [3,4,13,17]. In this situation, SD arises near the seizure-onset zone and then non-synaptically (3–9 mm/min velocity) propagates across the ipsilateral cortex [3]. A few studies have shown that generalized tonic-clonic seizures can induce SD in the cortex [15,19,20], but the seizures involve bilateral cortical and subcortical networks of the brain. Therefore, we hypothesize that generalized tonic-clonic seizures can trigger SD multifocally. To examine the suggestion, we studied the occurrence of SD in the cortex and hippocampus during acute tonic-clonic seizures, using the pentylenetetrazole (PTZ) model and in vivo electrophysiology in awake animals. Pentylenetetrazole, a non-competitive antagonist of GABA(A) receptors, induces tonic-clonic seizures by producing an excitation/inhibition imbalance. Systemic administration of PTZ uniformly suppresses GABA inhibition and reduces the inhibitory tone throughout the brain, which mimics a global defect of GABAergic signaling associated with generalized tonic-clonic seizures. EEG patterns of the PTZ-induced seizures closely match those seen in human generalized epilepsies. Our findings show that generalized tonic-clonic seizures rapidly trigger SD waves in many regions of the cortex and hippocampus.

## 2. Results

### 2.1. Generalized Tonic-Clonic Seizures Are Accompanied by Widespread Triggering of SD

**Figure 1:**
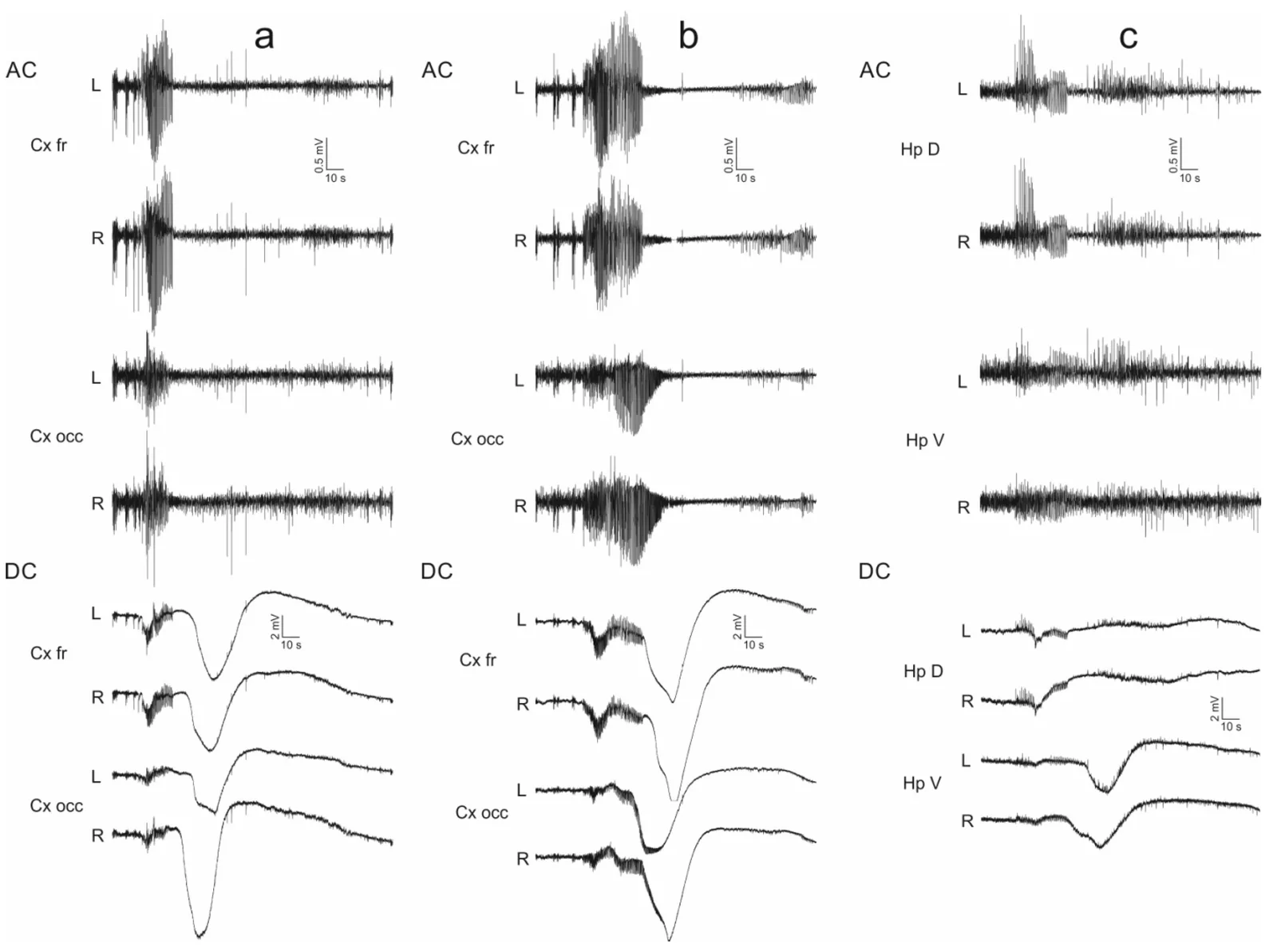
Generalized tonic-clonic seizures trigger SD in the cortex and hippocampus of awake rats. Representative recordings of three seizure episodes recorded in the cortex (**a**,**b**) and hippocampus (**c**). Ictal activity and seizure-induced SD were recorded in the frontal (Cxfr) and occipital (Cx occ) cortices (**a**,**b**) and in the dorsal (HpD) and ventral (HpV) hippocampi (**c**) of the left (L) and right (R) hemispheres. Upper and lower fragments show AC (1–100 Hz) and DC (0–100 Hz) recordings, respectively, of the same seizure episode. SD could be detected in DC recordings only.

Figure 1 shows AC (upper fragments) and DC (lower fragments) patterns of PTZ-induced seizures detected in the cortex (a, b) and hippocampus (c).

**Figure 2:**
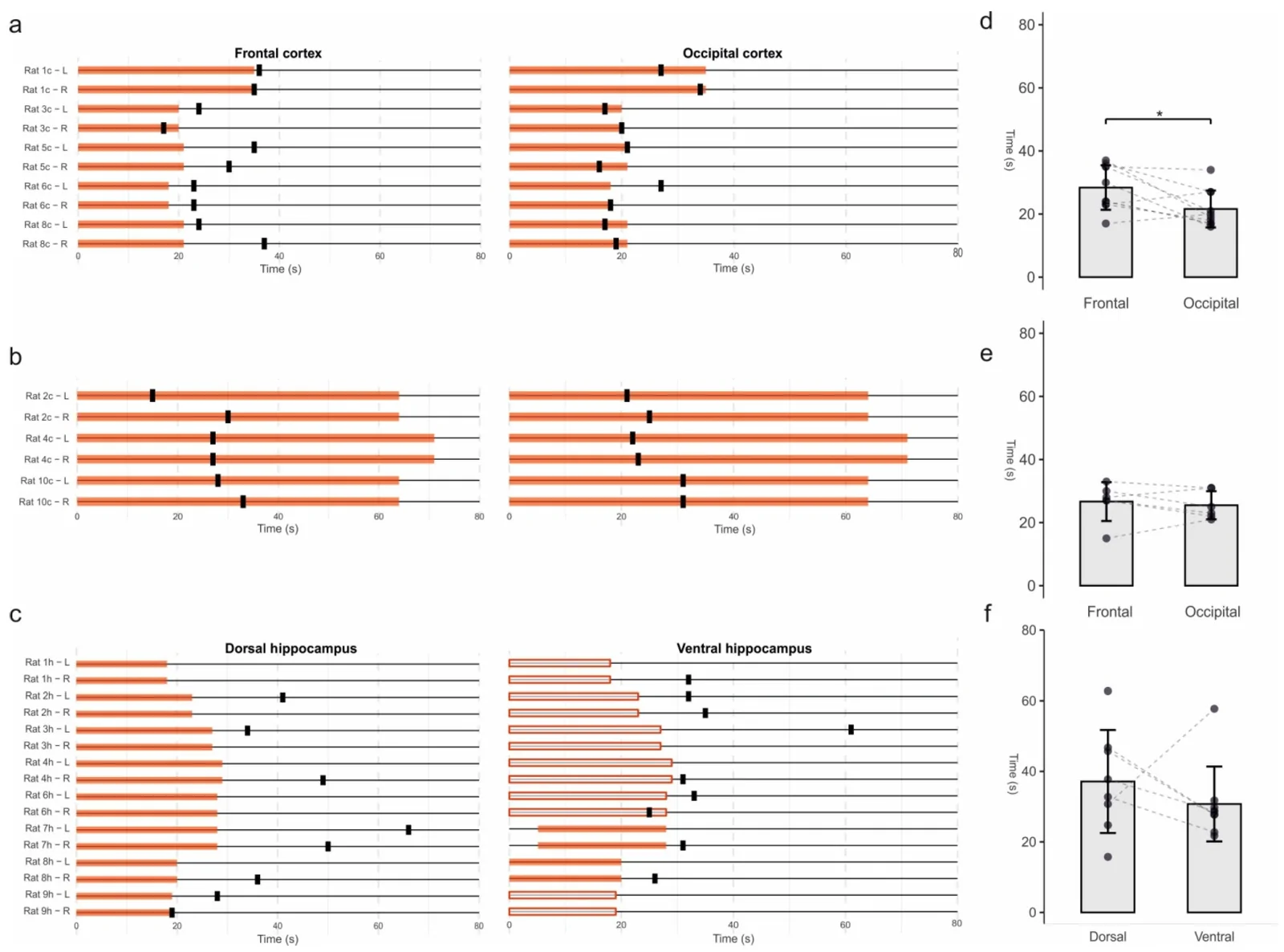
The latency of SD appearance in the cortical and hippocampal regions after generalized tonic-clonic seizures. (**a**–**c**)—individual data on seizure duration (red lines) and SD onset (black marks) in the frontal/occipital cortex (**a**,**b**) and dorsal/ventral hippocampus (**c**). Lines show the timelines for the events detected in the left (L) and right (R) hemispheres of each rat with cortical (Rats 1c–10c) and hippocampal (Rats 1h–9h) recordings. The ventral hippocampus frequently remained uninvolved in epileptic activity (white lines mark the corresponding ictal period in the dorsal hippocampus). (**d**–**f**)—the mean latency of cortical SD (the period from the seizure start to SD onset) in each region after brief (**d**) and long (**e**) seizures and the mean latency of hippocampal SD (**f**). After brief seizures (**d**), SD appeared significantly earlier in the occipital cortex than in the frontal cortex (*p* = 0.04165, pairwise *t*-test). * *p* < 0.05.

Administration of a single dose of PTZ (50 mg/kg) induced tonic-clonic seizures in 8 of 9 rats equipped with cortical recording electrodes. As seen in AC traces (Figure 1a,b, upper fragments), the ictal discharge represented high-voltage spike/spike-wave activity followed by postictal EEG depression. Seizures were either brief (25.6 ± 5.6 s, range 18–48 s, *n* = 5) or long (66.3 ± 2.3 s, range 64–71, *n* = 3). Direct current (DC) recordings of the same seizure episodes (Figure 1a,b, lower fragments) showed that a single SD wave (a negative high-amplitude potential shift) occurred nearly simultaneously in all cortical regions of the two hemispheres. In all rats (*n* = 8), SD appeared in close temporal association with the ictal discharge. Individual data on the occurrence of seizure-induced SD for each channel are presented in Table S1 and Figure 2a,b.

The same dose of PTZ induced brief tonic-clonic seizures in 8 of 9 rats with hippocampal recording electrodes. The seizure episodes lasted 23.1 ± 1.9 s (range 16–29 s, *n* = 8) and represented bursts of high-voltage positive spikes followed by spike-wave activity (Figure 1c, upper fragment). The ictal activity was detected in the dorsal hippocampus of all (8/8) rats and in the ventral hippocampus of only 2 of 8 rats. As in the cortex, the generalized seizure was followed by hippocampal SD and a brief depression of ongoing activity (Figure 1c). Unlike the cortex, hippocampal SD had low amplitude and never appeared in all channels (Figure 1c, lower fragment). In total, 16 LFP recordings were obtained from both sides of the hippocampus in 8 rats. SD was detected in the dorsal and ventral hippocampus after 50% (8/16) and 56% (9/16) of seizures, respectively. The probability of post-seizure SD was significantly lower in the hippocampus than that in the cortex (*p* = 0.0133 for dorsal and *p* = 0.0077 for ventral hippocampus, McNemar test). Individual data on seizure-induced hippocampal SD are presented in Table S2.

Thus, SD occurred in all regions of the cortex and certain regions of the hippocampus in association with generalized tonic-clonic seizures.

### 2.2. Timing of Seizure-Induced Cortical and Hippocampal SD

Next, we studied the latency of SD appearance relative to the termination and onset of a seizure episode. Figure 2 shows individual data on the timing of seizures and SD in each region of the cortex and hippocampus.

In the cortex, SD emerged significantly earlier in its occipital regions than in the frontal ones (*p* = 0.0417, pairwise *t*-test, Figure 2d). Brief seizures triggered SD at 5.4 ± 6.0 s (*n* = 10) after the seizure termination in the frontal cortex and at 1.4 ± 4.5 s (*n* = 10) before the seizure end in the occipital cortex (Figure 2a). Unexpectedly, we found that SD appeared with a similar delay after the onset of both brief and long seizures—at 28.4 ± 7.1 s and 26.7 ± 6.2 s, respectively, in the frontal cortex (*p* = 0.6154) and at 21.6 ± 5.9 s and 25.5 ± 4.5 s in the occipital cortex (*p* = 0.2618, *n* = 6, unpaired *t*-test, Figure 2b,e).

In the dorsal and ventral parts of the hippocampus, SD was detected after a delay of 16.3 ± 11.5 s (*n* = 8) and 9.1 ± 10.7 s (*n* = 9), respectively, after seizure termination and 40.4 ± 14.6 s and 34.0 ± 10.6 s after seizure onset (Figure 2c,f). The hippocampus showed no regional difference in the SD latency (*p* = 0.9466, GEE method). Though the ventral hippocampus often remained uninvolved in epileptic activity, short-latency SDs appeared here with the same probability and latency as in the dorsal hippocampus expressing robust seizure activity (Figure 2c,f).

Thus, SD appears within the first 20–40 s of ictal activity in all regions of the cortex and hippocampus.

### 2.3. Postictal Depression and Seizure-Induced SD

**Figure 3:**
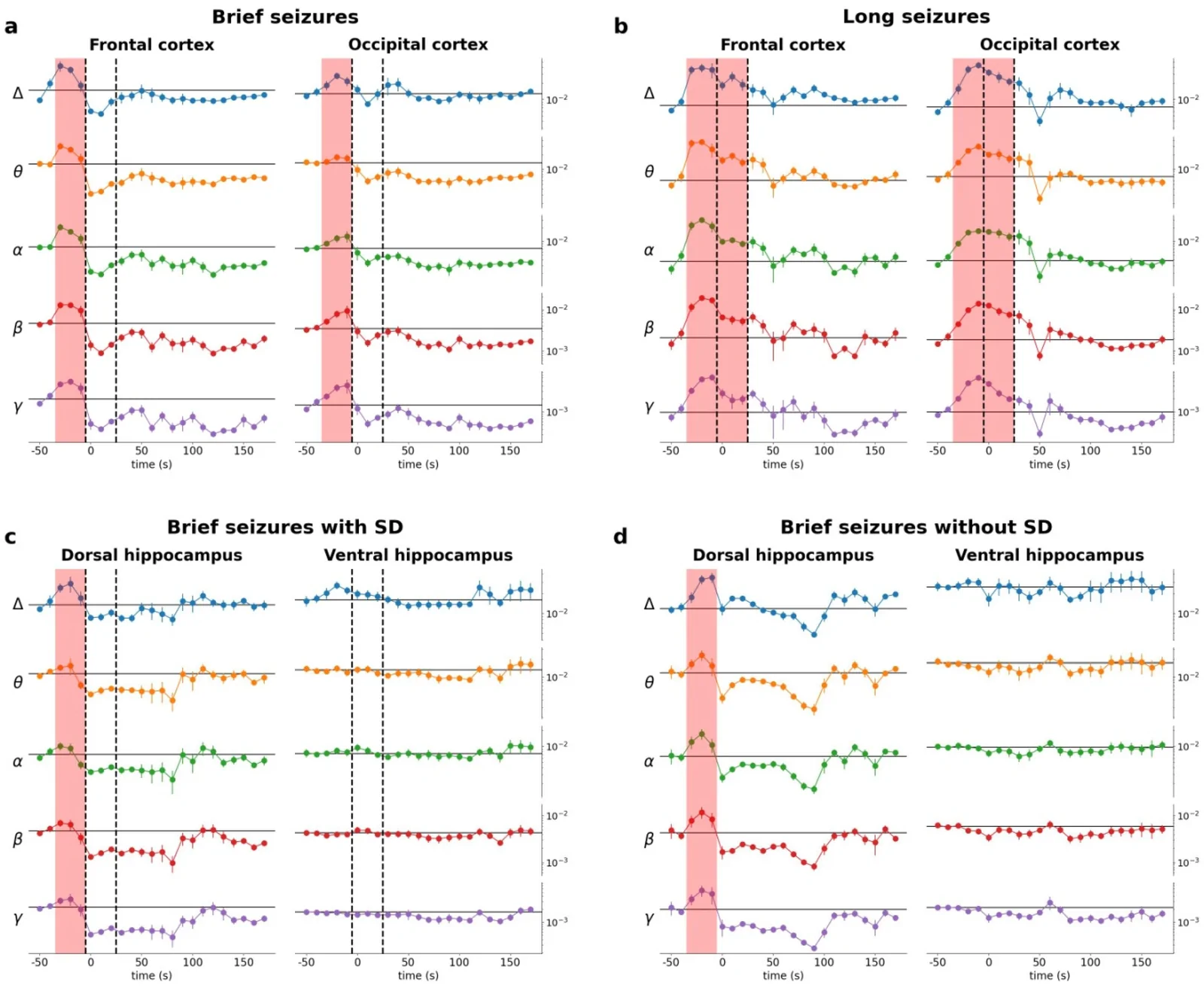
Ictal and postictal changes in the power of cortical and hippocampal activity induced by generalized tonic-clonic seizures in awake rats. The graphics show the mean log of power for Δ (1–4 Hz), θ (4–8 Hz), α (8–12 Hz), β (12–25 Hz), and γ (25–50 Hz) frequency bands (marked on the left *Y*-axis) in the frontal and occipital cortices after brief (**a**) and long (**b**) tonic-clonic seizures and in the dorsal and ventral hippocampus after seizures with (**c**) and without (**d**) SD. Within each band, horizontal lines mark the pre-seizure level, and circles show the mean power for 10-s intervals before (−50–0 s) and after (0–180 s) the SD onset. Red vertical areas in each fragment mark the seizure; dashed vertical lines show the onset (point 0) and offset of seizure-induced SD.

It is thought that SD terminates seizures by silencing neuronal activity [3]. We examined whether the occurrence of seizure-induced SD was accompanied by postictal depression. Using spectral analysis, we evaluated ictal and postictal changes in cortical and hippocampal activity. Expectedly, the power of spontaneous activity in the cortex and dorsal hippocampus increased during generalized tonic-clonic seizures (−30–0 s, Figure 3). In the cortex, brief seizures, terminated by SD, were followed by pronounced depression of cortical activity that started during SD (0–30 s) and persisted after its termination (Figure 3a). The postictal depression involved both cortical regions and all frequency bands except delta. If cortical seizures were long and seizure-induced SD started during the ictal episode, only mild beta-gamma depression was observed during the postictal period (Figure 3b). In the dorsal hippocampus, brief postictal depression developed, irrespective of SD occurrence (Figure 3c,d). In the ventral hippocampus, which was not recruited in the ictal discharges, SD did not change ongoing activity (Figure 3c).

Thus, in the cortex, brief generalized seizures culminate in cortical SD with pronounced postictal EEG depression, while in the hippocampus, the occurrence of SD does not correlate with postictal depression.

### 2.4. Characteristics of Cortical and Hippocampal SDs Induced by Seizures and Mechanical Stimulation

**Figure 4:**
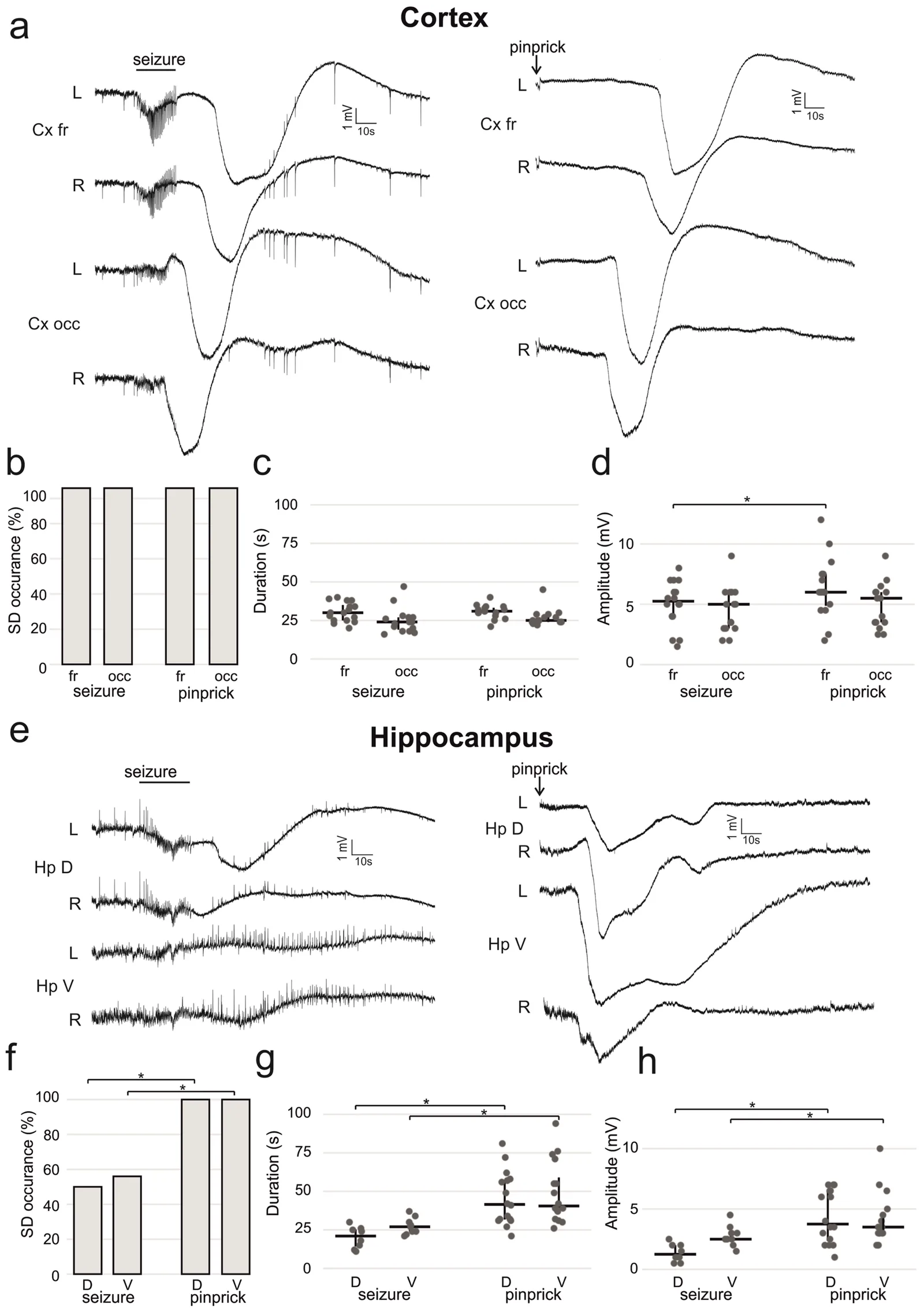
Comparison of cortical and hippocampal SDs induced by a seizure and a pinprick. (**a**)—representative recordings of SDs induced by a seizure (left fragment, the seizure is marked by a line) and a pinprick of the somatosensory cortex (right fragment, the pinprick is marked by an arrow) in the same rat and recorded in the frontal (Cx fr) and occipital (Cx occ) cortices of the left (L) and right (R) hemispheres. (**b**–**d**)—the occurrence (**b**), duration (**c**), and amplitude (**d**) of SD induced by the seizure and the pinprick in the same rats. (**e**)—representative recordings of hippocampal SDs induced by a seizure (left fragment) and a pinprick of the dorsal hippocampus (right fragment) in the same rat and recorded in the left and right dorsal (HpD) and ventral (HpV) hippocampi. (**f**–**h**)—the occurrence (**f**), duration (**g**), and amplitude (**h**) of SD induced by the seizure and the pinprick. *—*p* < 0.05.

Next, we compared spatiotemporal characteristics of seizure- and mechanically induced SDs. Figure 4a–d shows the incidence and parameters of cortical SD triggered by a seizure and a pinprick in the same rats (a within-subject control). In all rats, both the seizure and mechanical stimulation induced SD that spread to all regions of the cortex (Figure 4a,b, Table S1). The duration of seizure- and mechanically induced SDs was similar in both the frontal (*p* = 0.9645) and occipital (*p* = 0.2266) cortex (Wilcoxon test, Figure 4c). The amplitude of SD induced by seizures was slightly lower than that of mechanically induced SD in the frontal cortex (*p* = 0.0469) but not in the occipital cortex (*p* = 0.1119, Wilcoxon test, Figure 4d). This confirms the high susceptibility of the cortex to SD, irrespective of the nature of the initiating stimulus.

In the hippocampus, seizures induced SD half as often as direct mechanical stimulation (*p* < 0.05, Figure 4e,f, Table S2). Moreover, the amplitude and duration of seizure-induced SD were significantly lower compared to those of pinprick-induced SDs (*p* = 0.0469 for the dorsal and ventral hippocampus, Wilcoxon test, Figure 4g,h).

Thus, both a pinprick and a seizure trigger a full-blown SD in the cortex, whereas a seizure induces an aborted, partially blocked SD in the hippocampus.

## 3. Discussion

We demonstrate that generalized tonic-clonic seizures rapidly trigger SD in many regions of the cortex and hippocampus. Everywhere, SD appears within the first 20–40 s of ictal activity, irrespective of its duration. Since the regions are remote from each other and some of them have no pathways for non-synaptic propagation of SD, the short-latency, near-simultaneous appearance of SD in the sites suggests its multifocal initiation. It is likely that a widespread increase in neuronal excitability during generalized tonic-clonic seizures favors the appearance of multiple generators of SD. This differs from focal seizures, during which SD originates near the epileptic focus and slowly (non-synaptically) propagates to the ipsilateral extrafocal regions [3]. Thus, depending on seizure type, SD can be induced in a single brain site (propagated SD) or multiple brain sites (non-propagated SD). In patients with drug-resistant epilepsy, both propagated and non-propagated SDs have been recorded [11]. A recent study reported that electroconvulsive therapy, used for treatment of severe psychiatric conditions, initiates generalized seizures and cortical SD [20]. Given our findings regarding the ability of generalized seizures to trigger SD in the hippocampus, it is tempting to speculate that the seizure-induced subcortical SDs may be involved in mechanisms underlying the effects of electroconvulsive therapy.

We show that cortical SD appears 21–28 s after the seizure onset, irrespective of the total seizure duration. Interestingly, in another model of generalized seizures, cortical SD develops with the identical delay (21–28 s) after the onset of flurothyl-induced seizures [19]. In hippocampal slices, seizure-induced SD emerges more rapidly—during the first 10–15 s of ictal activity [5]. It is commonly accepted that the initiation of SD depends on rising extracellular potassium concentrations in the course of the ictal episode [5,6,21]. If so, the potassium levels necessary for the initiation of the self-regenerative process of depolarization (SD) are reached very rapidly after the seizure onset (no more than 40 s). However, some studies found no correlation between [K+]o levels and the likelihood of triggering SD [4,22], suggesting a role for other factors, such as changes in chloride homeostasis or sodium/potassium channel activity [3,4,22].

The short duration of PTZ-induced seizures suggests that active mechanisms, rather than energy exhaustion, are responsible for their spontaneous termination. Seizure-induced SD has been proposed as one such mechanism [3,4]. Our study demonstrates that the termination of brief seizures always coincides with the onset of the depolarization phase of SD. It looks like SD curtails the ictal episode and starts postictal depression. Frequent occurrence of SD at the end of seizures has been reported in the cortex of humans, anesthetized animals, and cortical slices [3,9,19] and in the hippocampus of awake rats [4,23]. Seizures with cortical SD are shorter than those without SD [4,20]. The data support the idea that SD can act as a rapid brake mechanism in the epileptic cortex [3,4]. However, we also observed that sometimes seizures continued despite the onset of cortical SD (long seizures), indicating occasional failure of the rapid anti-ictal effect of SD.

The unexpected finding of the present study is that generalized tonic-clonic seizures trigger SD in a region-dependent manner: full-blown SD in the cortex but partially blocked and spatially limited SD in the hippocampus. The cortex and hippocampus are the brain regions most vulnerable to SD and epileptic activity [6]. Focal seizures can trigger SD both in the cortex [3] and hippocampus [4]. Given that a pinprick of the non-epileptic hippocampus elicits full SD propagated to all hippocampal regions, the observed resilience of the hippocampus to seizure-induced SD may result from the hyperexcitable state of hippocampal networks (baseline or PTZ-induced). Experiments in awake mice with mutant potassium channels show that the occurrence of SD after seizures strongly depends on intrinsic neuronal excitability [22]. In the core of the epileptic focus exhibiting intense and persistent epileptic activity, SD has reduced amplitude and duration, slower propagation speed, and low penetration rate [3,17]. Progressive enhancement of cortical excitability during PTZ kindling is accompanied by gradual suppression of seizure-induced cortical SD, with its transformation from a full-blown pattern (before kindling) to partially blocked SDs (at the middle kindling stages) and then to the complete blockade of SD occurrence at the final stages of epileptogenesis when epileptic activity becomes strong and persistent [16]. Disinhibition easily elicits SD in cortical slices from non-epileptic rats but cannot trigger SD in slices from chronically epileptic patients and rats [24]. Rare triggering of SD by epileptic activity has been reported in hippocampal slices [25]. We suggest that reduced inhibitory tone and hyperexcitability of hippocampal networks counteract the occurrence of SD in the hippocampus.

To sum up, generalized tonic-clonic seizures are followed by rapid and widespread triggering of SD in the cortex and hippocampus. Since detection of SD in patients is challenging, this information is important for understanding pathogenic mechanisms and consequences of generalized tonic-clonic seizures. A deeper mechanistic insight into the seizure–SD interplay may reveal novel therapeutic avenues for aborting prolonged ictal activity and managing refractory status epilepticus.

## 4. Materials and Methods

### 4.1. Animals

Experiments were performed in awake, freely behaving Wistar rats (300–380 g, Scientific Center for Biomedical Technologies of the Federal Medical and Biological Agency, Russia; males, *n* = 21). Animals were housed under temperature-controlled conditions (22 °C ± 2 °C) on a 12-h/12-h light/dark cycle with food and water available ad libitum. Experiments were designed and performed in accordance with the ARRIVE guidelines and Directive 2010/63/EU for animal experiments. The study protocol was approved by the Ethics Committee of the Institute of Higher Nervous Activity and Neurophysiology (protocol N1, 01.02.2022).

### 4.2. Surgery

Two weeks before the start of the experiment, bilateral recording electrodes were implanted in the cortex (10 rats) or hippocampus (10 rats) under isoflurane anesthesia. Cortical electrodes were positioned in the frontal (AP: +1.2 mm, ML: ± 2.3 mm, DV: −1.5 mm) and occipital (AP: −5.9 mm, ML: ± 3.5 mm, DV: −1.5 mm) cortex of the two hemispheres. Hippocampal electrodes were implanted in the dorsal (AP −2.3 mm, ML ± 1.6 mm, DV 3.7 mm) and ventral (AP −6 mm, ML ± 4.9 mm, DV 6 mm) parts of the left and right hippocampi. A reference electrode was placed over the cerebellum. Guide cannulas (23 gauge) for mechanical initiation of SD were positioned in either the somatosensory cortex (AP −2.8 mm, ML ± 4.5 mm, DV 1.5 mm) or the dorsal hippocampus (AP −4.4 mm, ML ± 3.9 mm, DV 2.5 mm) in rats with cortical or hippocampal recording electrodes, respectively. Recording electrodes, cannulas, and pin connectors were fixed on the skull with acrylic dental plastic. During the post-surgery period, rats were injected with the antibiotic amoxicillin (0.3 mL, i.m.) and glucose solution (1 mL, s.c.). Experiments started two weeks after the surgery. In total, 18 rats were used in the experiments (two rats died during the post-surgery period).

### 4.3. Experimental Design

Rats were individually placed in a shielded chamber, and full-band electrical activity (0–100 Hz, 1 kHz sampling rate) of the cortex/hippocampus was recorded using a four-channel, high-input impedance (1 gΩ) dc amplifier and a/d converter (E14-440, L-Card, Moscow, Russia) with simultaneous video-monitoring of behavior. To induce generalized tonic-clonic seizures, rats were injected with 50 mg/kg (i.p.) of pentylenetetrazole (PTZ, Sigma, St. Louis, MO, USA), a GABA(A) receptor antagonist. The dose of PTZ is known to produce clonic/tonic-clonic seizures in Wistar rats [26]. In our study, PTZ induced seizures in 16 of 18 animals with implanted electrodes (8 of 9 rats with cortical electrodes and 8 of 9 rats with hippocampal electrodes). In most rats (5/8 in the cortical group and 6/8 in the hippocampal group), PTZ provoked a single ictal episode; if several seizures occurred, only the first episode was included in the analysis. Because seizures were recorded in both hemispheres, 16 generalized-seizure recordings were obtained for each region (the frontal and occipital cortices, the dorsal and ventral hippocampus), with 8 from one hemisphere and 8 from the other. A week after the PTZ experiment, SD was induced in the same rats by a pinprick of the somatosensory cortex or dorsal hippocampus, as described previously [27]. Briefly, the needle was inserted into the guide cannula and protruded from its tip by 1.0 mm.

For offline analysis, full-band LFP recordings were filtered with a low-pass filter at 0–50 Hz (direct current, DC recording) and a bandpass filter at 1–50 Hz (AC recording). Seizures and SD were identified using AC and DC recordings, respectively. Ictal activity was determined as high-voltage (exceeding two standard deviations) rhythmic spike-wave/spike activity lasting at least 5 s and accompanied by tonic-clonic convulsions. SD was identified by a high-voltage negative DC potential shift. The latency of SD was defined as the period between a seizure onset/offset and the onset of DC shift at the recording electrode. The duration of SD was measured from the start of DC deflection to its return to 90% of the baseline level. The amplitude of SD was measured from the pre-SD baseline DC level to the maximal negative deflection. Analysis of SD parameters was performed by a researcher who was blinded to the experimental condition (mechanical stimulation or PTZ treatment).

### 4.4. Spectral Analysis

Spectral analysis was performed on 230 s artifact-free segments of LFP recordings. The analysis was carried out starting from 50 s before the onset of the SD (point 0 is the SD onset). The epochs were divided into non-overlapping 10 s intervals, and time-dependent changes in the mean power for each frequency band were evaluated. The segments were filtered using a Butterworth digital filter: a high-pass filter with a 1 Hz cutoff frequency. Spectral power was computed using a fast Fourier transform (FFT) routine for five frequency bands: delta (1–4 Hz), theta (4–8 Hz), alpha (8–12 Hz), beta (12–25 Hz), and gamma (25–50 Hz). For each frequency band, the mean absolute power per interval was calculated.

### 4.5. Histological Analysis

After the end of the experiment, animals were deeply anesthetized and perfused intracardially with 0.9% saline. The brains were removed, stored in 10% formalin for 48 h, sectioned into 50-μm coronal slices, and stained with 0.1% cresyl violet. The slices were used for histological verification of the locations of the recording electrodes and the SD initiation site (Figure S1).

### 4.6. Statistical Analysis

Statistical processing was carried out using programs written in R version 4.5.2 (https://cran.r-project.org). An appropriate group size was determined based on the previous experimental data. If the data distribution did not pass the normality test (Shapiro–Wilk test (shapiro.test() function)), the Wilcoxon signed-rank test for pairwise comparisons was used (wilcox.test (x, y, paired = TRUE)). The data on timing of cortical SD were distributed normally (Shapiro–Wilk test) and were compared using pairwise and unpaired *t*-tests adjusted for multiple comparisons with false discovery rate (FDR) correction using the Benjamini–Hochberg procedure. Since seizure-induced SD was detected only in some regions of the hippocampus, the data were compared using the generalized estimating equations (GEE) method with an exchangeable correlation structure, clustered by rat. The occurrence of cortical and hippocampal SD after seizures and pinpricks was compared using the McNemar test. The timing of hippocampal SD was analyzed based on regression in time using a gamma distribution with a log-link function (geeglm(rd_time ~ region * hemisphere, id = rats, data = df, family = Gamma(link = “log”), corstr = “exchangeable”). The duration and amplitude of cortical and hippocampal SD induced by seizures and mechanical stimulation in the same animal were compared using a Wilcoxon signed-rank test for pairwise comparisons with FDR correction for multiple comparisons. Data on SD parameters are shown as median ± interquartile range; other data are expressed as mean ± SEM with the significance threshold set at *p* < 0.05.

## Data Availability

The datasets generated and analyzed during the current study are available from the corresponding author upon reasonable request. The data are not publicluy available due to privacy restrictions.

## Supplementary Materials

The following supporting information can be downloaded at: https://www.mdpi.com/article/10.3390/ijms27188429/s1.
