# Supplementary material for: Multifocal Initiation of Spreading Depolarization by Generalized Tonic-Clonic Seizures
Source: Int J Mol Sci. 2026 Sep 21;27(18):8429. doi: 10.3390/ijms27188429 (PMC13607438; doi:10.3390/ijms27188429)
Supplement: Supplementary file 1 [file ijms-27-08429-s001.zip › Table S2.pdf]

Table S2. Individual data on the occurrence of SD in hippocampus after generalized tonic-clonicseizures and mechanical stimulationin freely-behaving rats.

Rat	Seizure-induced hippocampal SD				Mechanically induced hippocampal SD			
	Dorsal R	*Ventral R*	*Dorsal L*	*Ventral L*	*Dorsal R*	*Ventral R*	*Dorsal L*	*Ventral L*
1	-	-	-	SD	SD	SD	SD	SD
2	-	SD	SD	SD	SD	SD	SD	SD
3	-	-	SD	SD	SD	SD	SD	SD
4	SD	SD	-	-	SD	SD	SD	SD
6	-	SD	-	SD	SD	SD	SD	SD
7	SD	SD	SD	-	SD	SD	SD	SD
8	SD	SD	-	-	SD	SD	SD	SD
9	SD	-	SD	-	SD	SD	SD	SD

Abbreviations: R, right; L, left; SD – detection of spreading depolarization in the regions.
