# Supplementary material for: Multifocal Initiation of Spreading Depolarization by Generalized Tonic-Clonic Seizures
Source: Int J Mol Sci. 2026 Sep 21;27(18):8429. doi: 10.3390/ijms27188429 (PMC13607438; doi:10.3390/ijms27188429)

**Figure S1.** Representative histological images showing localization of recording electrode in the occipital cortex (a) and guide cannula in the dorsal hippocampus (b). Staining with cresyl violet, scale bars = 0.3 mm (a) and 0.5 mm (b).

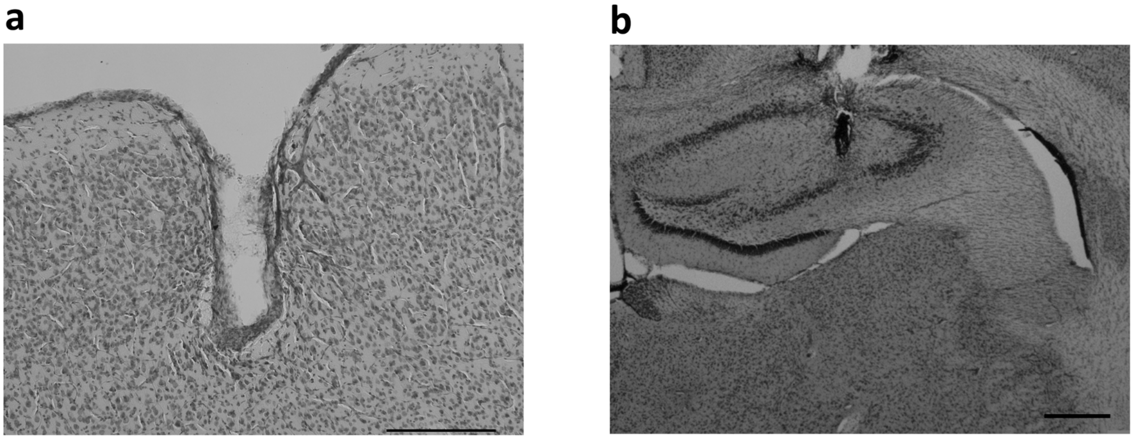

Supplement: Supplementary file 1 [file ijms-27-08429-s001.zip › Figure S1.pdf]
