# Supplementary material for: Multifocal Initiation of Spreading Depolarization by Generalized Tonic-Clonic Seizures
Source: Int J Mol Sci. 2026 Sep 21;27(18):8429. doi: 10.3390/ijms27188429 (PMC13607438; doi:10.3390/ijms27188429)
Supplement: Supplementary file 1 [file ijms-27-08429-s001.zip › Table S1.pdf]

Table S1. Individual data on the occurrence of cortical SD after generalized tonic-clonicseizures and mechanical stimulation in freely-behaving rats.

Rat	Seizure-induced cortical SD				Mechanically induced cortical SD			
	Frontal R	*Occipital R*	*Frontal L*	*Occipital L*	*Frontal R*	*Occipital R*	*Frontal L*	*Occipital L*
1	SD	SD	SD	SD	SD	SD	SD	SD
2	SD	SD	SD	SD	SD	SD	SD	SD
3	SD	SD	SD	SD	SD	SD	SD	SD
4	SD	SD	SD	SD	SD	SD	SD	SD
5	SD	SD	SD	SD	SD	SD	SD	SD
6	SD	SD	SD	SD	SD	SD	SD	SD
8	SD	SD	SD	SD	SD	SD	SD	SD
10	SD	SD	SD	SD	SD	SD	SD	SD

Abbreviations: R, right; L, left; SD – detection of spreading depolarization in the regions.
